# Fundamentals and Applications of ZnO-Nanowire-Based Piezotronics and Piezo-Phototronics

**DOI:** 10.3390/mi14010047

**Published:** 2022-12-25

**Authors:** Yitong Wang, Wanli Xie, Wenbo Peng, Fangpei Li, Yongning He

**Affiliations:** 1 School of Microelectronics, Xi’an Jiaotong University, Xi’an 710049, China; 2 The Key Lab of Micro-Nano Electronics and System Integration of Xi’an City, Xi’an 710049, China; 3 State Key Laboratory of Solidification Processing, Key Laboratory of Radiation Detection Materials and Devices, School of Materials Science and Engineering, Northwestern Polytechnical University, Xi’an 710072, China

**Keywords:** piezotronic effect, piezo-phototronic effect, ZnO nanowires, sensors, optoelectronic devices, energy harvesting

## Abstract

The piezotronic effect is a coupling effect of semiconductor and piezoelectric properties. The piezoelectric potential is used to adjust the p-n junction barrier width and Schottky barrier height to control carrier transportation. At present, it has been applied in the fields of sensors, human–machine interaction, and active flexible electronic devices. The piezo-phototronic effect is a three-field coupling effect of semiconductor, photoexcitation, and piezoelectric properties. The piezoelectric potential generated by the applied strain in the piezoelectric semiconductor controls the generation, transport, separation, and recombination of carriers at the metal–semiconductor contact or p-n junction interface, thereby improving optoelectronic devices performance, such as photodetectors, solar cells, and light-emitting diodes (LED). Since then, the piezotronics and piezo-phototronic effects have attracted vast research interest due to their ability to remarkably enhance the performance of electronic and optoelectronic devices. Meanwhile, ZnO has become an ideal material for studying the piezotronic and piezo-phototronic effects due to its simple preparation process and better biocompatibility. In this review, first, the preparation methods and structural characteristics of ZnO nanowires (NWs) with different doping types were summarized. Then, the theoretical basis of the piezotronic effect and its application in the fields of sensors, biochemistry, energy harvesting, and logic operations (based on piezoelectric transistors) were reviewed. Next, the piezo-phototronic effect in the performance of photodetectors, solar cells, and LEDs was also summarized and analyzed. In addition, modulation of the piezotronic and piezo-phototronic effects was compared and summarized for different materials, structural designs, performance characteristics, and working mechanisms’ analysis. This comprehensive review provides fundamental theoretical and applied guidance for future research directions in piezotronics and piezo-phototronics for optoelectronic devices and energy harvesting.

## 1. Introduction

Energy has always been a popular research subject. The effective utilization of solar energy and mechanical energy (e.g., vibration energy, wind energy, and water/blue energy) has attracted great research interest. Emerging devices that effectively harvest different kinds of energies and further efficiently convert them into electricity in various forms deserve thorough research. Solar cells, for example, can function as an electric source by providing an open-circuit voltage or a short-circuit current by absorbing solar light from sunshine. As a sustainable energy source to replace fossil energy sources, it has become the main development direction in the industrial field [1]. A light-emitting diode (LED) is a cold light source that can emit photons with different wavelengths and has experienced very important development in the field of biosensing and display [2,3]. In addition to optoelectronic devices, including solar cells, LEDs, and photodetectors (one kind of optoelectronic device is similar to solar cells but usually used for the detection of photons), electronic devices such as transistors and sensors are fundamental basics of logic computation, integrated circuit, and Internet of Things, which are of great significance in our modern society. Among them, piezoelectric transistors are devices that use the externally applied strain as a gate to control the carrier transport process and are widely used in the fields of logic operations and tactile sensing [4,5,6]. Since the dominant working mechanism of most optoelectronic devices depends on the transport characteristics of photo-generated carriers through interfaces inside the space charge region of junctions, e.g., p-n junctions and Schottky junctions, improving optoelectronic devices’ energy-conversion efficiency can be realized by enhancing carrier transport abilities by modifying the properties of space charge region near the interface. Therefore, it is desirable to improve the energy-conversion efficiency for energy harvesting and photoelectric sensing. Piezoelectric transistors utilizing the externally applied strain as the gate to control carrier transport processes have the greatest potential, which indicates that the utilization of mechanical strains in piezoelectric semiconductor-material-based electronic and optoelectronic devices is a possible methodology.

The piezoelectric effect refers to generating a piezoelectric potential/voltage when materials with non-centrosymmetric crystal structures are deformed. This is a phenomenon in which mechanical energy and electrical energy are exchanged in both directions, i.e., mechanical energy can be converted into electrical energy through the piezoelectric effect; meanwhile, electrical energy can also be converted back into mechanical energy through the inverted piezoelectric effect. In 2006, Prof. Zhong Lin Wang first demonstrated the fundamental principle of piezotronics, which utilizes the coupled piezoelectric and semiconducting properties of nanowires and nanobelts for designing and fabricating electronic devices, such as field-effect transistors and diodes [7]. In more detail, the piezotronic effect is the two-way coupling effect of the piezoelectric effect and the semiconducting characteristics, which uses the interface polarization charge caused by the strain to modulate the energy band structure at the interface and then effectively control the transportation of the carriers at the interface or junction region [8]. At present, it has been widely used in strain-gated transistors [9,10,11], electronic skin [12], strain sensors [13], tactile imaging [14], and other fields. In 2010, Prof. Zhong Lin Wang further demonstrated piezo-phototronics in ZnO micro/nanowire devices [15]. The piezo-phototronic effect is the three-field coupling of semiconductor, photoexcitation, and piezoelectric properties using the piezoelectric potential to adjust/modulate and control the generation, separation, transport, and other recombination processes of carriers in the interface and junction regions, which can realize high-performance optoelectronic devices with novel features [16].

Among the commonly seen piezoelectric semiconductor materials, one-dimensional ZnO nanowires (NWs) are one of the best materials for studying the piezotronic and piezo-phototronic effects. The main reason is that the growth methods of ZnO NWs are diverse, facile, and low-cost. Low-defect and high-quality ZnO NWs can be grown in a high-temperature tube furnace using a vapor–solid process. Moreover, a uniform ZnO NWs array can also be grown using low-temperature hydrothermal methods [17,18]. At the same time, ZnO NWs can grow on almost any material and shape-based substrate [19]. In addition, the biocompatibility of ZnO is perfect. Trace amounts of ZnO can even be absorbed by humans, making it an excellent choice for preparing flexible and biological-signal-driven electronic devices [20].

As piezotronics and piezo-phototronics have been studied and developed for many years since their creation in 2007 and 2010, respectively, it is of great importance to overview the fundamentals and applications of piezotronics and piezo-phototronics in recent years, especially for ZnO-nanowire-based electronic and optoelectronic devices. Herein, we selected ZnO with piezoelectric and semiconducting properties as the object in this review. The content mainly consisted of the current progress of piezotronic and piezo-phototronic effects based on ZnO nanowires. First, the growth methods for synthesizing n-type and p-type ZnO thin films and NWs on different substrates were summarized. Next, the mechanism and analysis of the piezotronic effect based on ZnO nanowires for sensor performance enhancement, multi-type energy harvesting, logic operation of piezo-gated transistors, and pollutant degradation in biochemical fields were summarized. Then, the piezo-phototronic effect used in photodetectors with different structures for different light wavelengths, the brightness enhancement of LEDs, and the external quantum efficiency improvement of solar cells were also summarized and compared. Finally, we presented a perspective regarding the possible future research directions and applications of piezotronics and piezo-phototronics. This systematic review hopes to provide a reference for fundamental and application research on piezotronics and piezo-phototronics in the fields of energy and human–machine interaction.

## 2. Growth Methods of ZnO Nanowires

Wurtzite crystalline ZnO NWs have excellent piezoelectric properties. To obtain better piezoelectric properties, growing better-quality ZnO NWs has also become a focus of research [20]. For ZnO NW devices, commonly used substrates include materials such as Si [21], SiC [17], GaN [22], sapphire [23], and flexible substrates [24].

There are three main methods adopted in the laboratory preparation of ZnO NWs. The first is the physical vapor deposition (PVD) method [25], also well known as the vapor–liquid–solid (VLS) process. ZnO and graphite are mixed and placed in a tube furnace. Argon (Ar) is used as the carrier gas, and oxygen is used to facilitate the reaction. A thin layer of gold is also applied to the substrate as a catalyst. The raw material is then placed in a tube and heated to about 900 °C. This method allows the growth of ZnO NWs with fewer defects, but the higher temperature is not suitable for all substrates.

The second method is metal–organic chemical vapor deposition (MOCVD) [26]. This method starts with the preparation of patterns using photolithography on a substrate, followed by the evaporation of 30Å of gold and exfoliation. ZnO NWs are, therefore, selectively grown on the Au patterns using MOCVD at 850–900 °C using Ar as the carrier gas and diethylzinc (DEZn) and oxygen as the reactant source. A similar growth method but without catalyst gold is also available [27]. This method also allows the growth of high-quality ZnO NW single crystals, but the reaction temperature also reaches about 900 °C.

The last method is the hydrothermal growth method [18,28,29]. The substrate is first magnetron-sputtered to deposit a seed layer of ZnO crystals [30] or a catalyst Au layer, and then the substrate is placed in a growth solution configured with zinc nitrate hexahydrate and hexamethylenetetramine (HMTA) for about one hour, and the substrate is placed face-down on top of the growth liquid. This method can change the aspect ratio, density, and shape of the ZnO NWs when over-controlling the hydrothermal reaction parameters, such as the concentration of the reaction solution, reaction time, and temperature. Hydrothermal growth conditions are simple and less expensive, with low temperatures, and can be applied to almost all substrates.

In addition, Wang et al. investigated the preparation of high-quality ZnO NWs at the wafer level. Periodic nanopatterns were prepared by laser interference lithography (LIL) [31] and the pulsed laser interference ablation (LIA) method [32] with fewer process steps. Then, low-temperature hydrothermal growth was performed to obtain ZnO NWs. This method has the advantages of being large-scale, rapid, mask less, and non-contact and is well-suited for the large-scale industrial preparation and integration of ZnO NWs. ZnO NWs are generally grown perpendicular to the substrate interface [21], but laterally aligned arrays of ZnO NWs grown parallel to the substrate interface are also available [19,33] and are suitable for large-scale production, in addition to ZnO NWs of exceptional length for special needs [25].

## 3. Piezotronics Based on ZnO Nanowires

The core idea of the piezotronic effect is to use the piezoelectric potential to adjust the carrier transport characteristics. The piezoelectric potential generated when ZnO NWs are bent can act as a stress switch. This kind of device driven by mechanical deformation action is called a piezotronics device, which can be used to regulate the performance of sensors [34,35,36] and prepare piezoelectric diodes and piezoelectric field effect transistors [6,37,38,39].

### 3.1. Theoretical Research of the Piezotronic Effect

**Figure 1:**
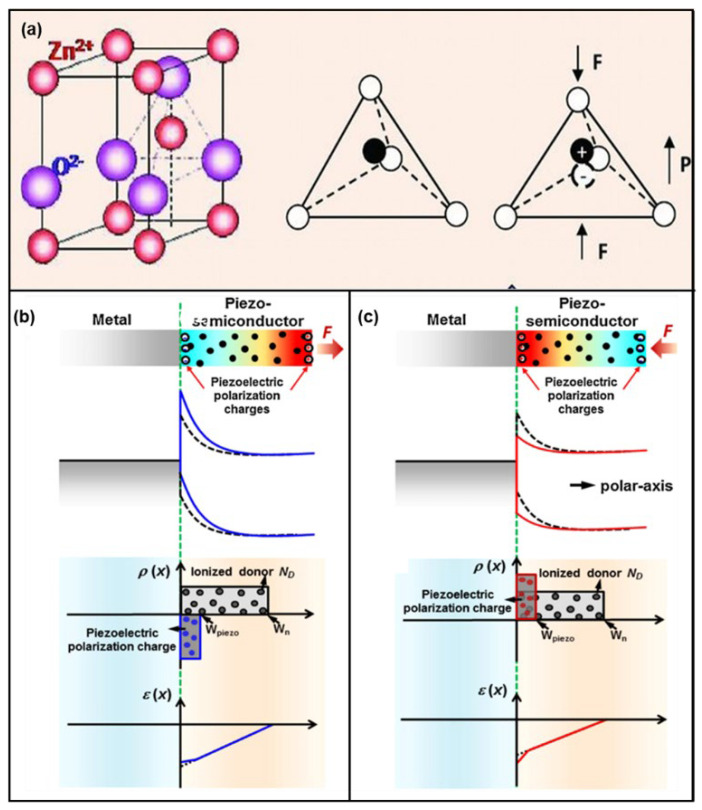
(**a**) Atomic model of the wurtzite-structured ZnO. Reproduced with permission from [40]. Copyright (2012) *Advanced Materials*. The energy band diagram, spatial charge distribution, and electric field schematics where (**b**) tensile strain is applied and (**c**) compressive strain is applied. Reproduced with permission from [61]. Copyright (2013) *Nano Today*.

ZnO has a non-centrosymmetric structure, as shown in Figure 1a. The O^2−^ anion is hexagonally packed, and the Zn^2+^ cation is filled in the middle of the tetrahedron formed by the anion [40]. When ZnO is not subjected to stress, the charge centers of the cation and anion in the tetrahedron coincide. When ZnO undergoes compressive deformation, the charge centers of the cations and anions are displaced relative to each other, creating an electric dipole moment. The sum of the dipole moments generated by all cells in the crystal generates a piezoelectric field, resulting in a macroscopic potential drop along the stress/strain direction in the crystal, called the piezoelectric potential. ZnO generally has piezoelectricity along the *c*-axis, which is usually the growth direction of ZnO NWs.

To study ZnO piezoelectric semiconductors’ nanostructural morphology, Wang et al. invented the piezoelectric nanogenerator of ZnO using the piezoelectric properties of ZnO NW arrays for the first time in 2006 [41]. In the same year, Wang used the piezoelectric potential generated by the strain of ZnO to regulate the electrical output of single ZnO NW devices and introduced the concept of piezotronics [42]. In the following decade, progress was made in developing ZnO piezoelectronic devices [43,44]. This has led to promising applications in sensors [13,45,46,47,48,49], strain-gated transistors [10,39,50,51,52], biochemistry [53,54], and energy harvesting [55,56,57,58,59].

The piezotronic effect is an effect resulting from the coupling of semiconductor and piezoelectric properties in a piezoelectric semiconductor material, which is a process that uses the piezoelectric potential (or piezoelectric charge) generated by a strain (compressive or tensile strain) in a piezoelectric semiconductor to modulate the transport properties of carriers in the piezoelectric semiconductor [60].

The theory of the piezotronic effect of p-n junctions and Schottky junctions was systematically compiled by Zhang et al. [61]., using the Schottky junction as an example [62], as shown in Figure 1. The Fermi energy level of the semiconductor will align with the Fermi energy level of the metal and will cause the internal charge of the device to rearrange when a metal and a semiconductor are in contact. Because of the difference in the work function and electron affinity energy of metals and semiconductors, the metal–semiconductor interface can form an Ohmic contact with linear resistance or a Schottky contact with rectification characteristics. Negative polarization charges are generated at the interface by applying compressive strain to the device, which is reflected in the energy band as an increase in the potential barrier in Figure 1b. Corresponding to the applied tensile strain, positive polarization charges are generated at the interface that lower the potential barrier, as illustrated in Figure 1c. According to the current diffusion theory and the electrically neutral equilibrium condition, the current flowing through the Schottky junction when there are piezoelectric charges generated at the interface can be obtained as follows [61]:

(1) $$J\approx J_{D0}\cdot \exp\left(\frac{q^{2}\;\rho_{\mathrm{piezo}}W_{\mathrm{piezo}}{}^{2}}{2{\mathrm{kT}\varepsilon }_{s}}\right)\cdot \exp\left[\frac{\mathrm{qV}}{\mathrm{kT}}-1\right]$$

where $J_{D0}$ is the saturation current density in the absence of piezoelectric charges, $\rho_{\mathrm{piezo}}$ is the density of piezoelectric polarization charges, and $W_{\mathrm{piezo}}$ is the depth of distribution of polarization charges at the device interface. It can be clearly seen that the current of the Schottky contact is efficiently regulated by the piezoelectric polarization charges.

Similar findings are found in the p-n junction. The current in the p-n junction is also an exponential function of the piezoelectric charge, the polarity of which depends on the strain (stretching or compression). Thus, the current can be effectively tuned not only by the magnitude of the strain but also by the stretching or compression of the device [61]. Zhang et al. also further investigated the modulation of the piezoelectric polarization charges using the theory of heterojunctions [63]. Luo et al. performed theoretical calculations on piezoelectric PIN diodes to improve the device system of piezoelectric electronics [64]. Jin et al. advanced their work to the small-signal dynamic characterization of the piezotronic effect by studying the diffusion capacitance and conductivity of piezoelectric p-n junctions under external compressive stress at low and high frequencies [65]. Furthermore, the researchers also simulated the piezotronic effects in more complex devices, such as FET [66], MIS devices [67], and BJT [68].

In addition to studying the controlling role of piezoelectric polarization charge at the interface, researchers have also investigated the more fundamental ZnO NW [69,70,71]. In 2010, Shao et al. proposed a continuum model for piezoelectric potential generation in bent ZnO nanorod cantilevers using the first piezoelectric effect approximation [72]. Wang et al. used scanning photoelectron spectroscopy microscopy to directly visualize the piezoelectric potentials in a single bent ZnO microwire [73]. Hu et al. explored the temperature properties of n-type ZnO NWs and found that lowering the temperature resulted in a greatly enhanced piezotronic effect [74]. Pei et al. experimentally found that the piezotronic effect of ZnO weakens the modulation of the piezoelectric polarization charge and deteriorates the rectification characteristics under higher light intensity [75]. Xue et al. also investigated the effect of an external electric field on ZnO NW, where the piezoelectric effect was significantly suppressed when increasing the external electric field’s strength [76]. Araneo performed extensive theoretical derivations and calculations to investigate the piezoelectric potential of quasi-one-dimensional ZnO NW, concluding that the piezoelectric properties are better using tapered NWs [77], and further calculated the I-V characteristics [78] and the thermoelectric model of ZnO NW [79]. Furthermore, Araneo explored the influence of the nonlinear piezoelectric effect in the properties of semiconductors and found that the effect of positive and negative polarities on the piezoelectric charge stack region devices is significantly different, with negative polarities being more favorable [80].

### 3.2. The Piezotronic Effect in Sensors

As the positive and negative charge centers in ZnO crystals separate under applied stress or strain, a piezoelectric potential is generated. Based on the piezotronic effect, ZnO has received extensive attention in the field of sensors. The physical and chemical properties of ZnO allow it to be used as piezoelectric sensors [81,82,83], gas sensors [84,85], molecular sensors [86,87], and other sensors [34,88,89,90,91].

**Figure 2:**
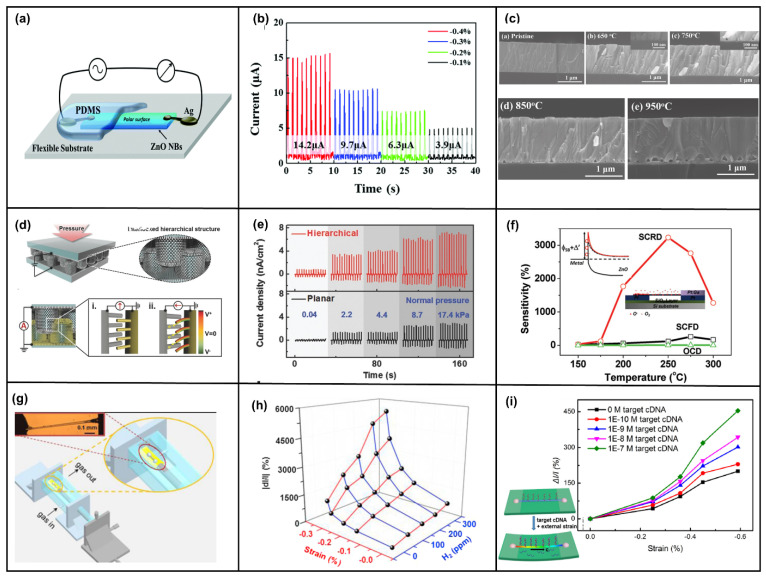
Device structure and the characterization of the In–ZnO nanoribbon (NB). (**a**) Device structure of the strain sensor of the In–ZnO NB. (**b**) The current response under different compressive strains. Reproduced with permission from [92]. Copyright (2015) *Nanoscale*. (**c**) Cross-sectional SEM images of as-grown and post-annealed ZnO thin film on Si at 650 °C, 750 °C, 850 °C, and 950 °C. Reproduced with permission from [94]. Copyright (2021) *Nano Energy*. Schematic diagram and electrical characteristics of device structure of bionic interlocking ZnO NW array. (**d**) Device structure and (**e**) current output density of nanogenerators. Reproduced with permission from [102]. Copyright (2015) *Advanced Functional Materials*. (**f**) The structure of Schottky’s contact ZnO NW gas sensor and sensitivity versus system temperature for O_2_ sensing. Reproduced with permission from [104]. Copyright (2009) *Journal of the American Chemical Society*. Schematic diagram and current test diagram of a piezoelectric effect enhanced nanowire gas sensor. (**g**) Schematic diagram of experimental equipment. Inset: optical image of a gas sensor device. (**h**) The amplitude of change in current under the action of hydrogen and pressure. Reproduced with permission from [105]. Copyright (2015) *Nano Energy*. (**i**) Relative current response under different compressive strains of Schottky-exposed ZnO NW DNA sensors. Inset: device structure. Reproduced with permission from [107]. Copyright (2016) *American Chemical Society Nano*.

The most common application of the piezotronic effect is sensors. A significant portion of strain sensors are made by connecting two electrodes on a substrate using ZnO NWs oriented along the *c*-axis. This structure requires attention to the polarization direction when applying pressure so that the wrong polarization does not degrade the device characteristics. Zheng et al. synthesized In-doped ZnO nanoribbons by means of chemical vapor deposition (CVD), and the polar orientation of the synthesized nanoribbons was <0001> perpendicular to the growth direction [92]. The structure is shown in Figure 2a, and the current response under different compression strains is shown in Figure 2b. The current change combines the piezoelectric effect, the Poisson effect, and semiconductor conductivity. The Schottky barrier heights (SBHs) of the source and drain electrodes in the special structure have the same trend under compressive and tensile strains, and the strain coefficient of the sensor reaches 4036 under compression.

In addition, many devices have ZnO NW perpendicular to the device’s cross-section. Self-powered pressure sensors of the double-sided ZnO NW configuration produce double the anisotropic piezoelectric voltage on both sides when bent, which is larger than just bent on one side [93]. To further improve the output of ZnO piezoelectric nanogenerators, ZnO films were obtained by annealing (650–950 °C) sputtering [94]. The film was a porous structure, as in Figure 2c, with the highest hole density at 850 °C. The output voltage was 7.5 times higher than the original ZnO (0.4 mV), the sensitivity was 6.9 times higher, and the piezoelectric coefficient (2.62 pm/V) was twice as high as the original ZnO (1.12 pm/V). In addition, the type of ZnO NW doping could be changed, and Sb-doped p-type ZnO NWs could be prepared using a hydrothermal method, improving the device structure [95]. Mechanical methods can also be used to transfer ZnO NWs [96]. The mechanical transfer process can advance device performance and take advantage of the high-quality NWs grown by methods such as high-temperature CVD.

Stretchable, wearable, self-powered flexible electronic devices are one of the mainstays of research in recent years [35,97,98,99,100]. Xu et al. proposed an initial self-powered flexible sensor based on n-ZnO/p-NiO [101]. The device was formed by magnetron-sputtering a layer of NiO on an ITO/PET substrate, followed by a layer of ZnO to form a heterojunction. The device received only 1% compressive strain, resulting in a current enhancement of about 200% and a sensitivity of 7.67 nA%^−1^ as a self-powered sensor. Min et al. reported the use of multi-level structured ZnO NW arrays to design electronic skin [102]. Layered ZnO NW arrays were first fabricated on polydimethylsiloxane (PDMS) microcolumns using a hydrothermal growth method and then coated with metal films to increase conductivity. The device structure is shown in Figure 2d. Due to the highly interlocking nanostructures, the applied pressure changed the contact area between the NWs, causing a change in contact resistance. The flexible electronic skin showed 3.7 times higher piezo-sensitivity (−6.8 kPa^−1^) and ultra-fast response time (<5 ms) compared to planar NWs, and could also detect acoustic pressure (≈57 db). The multi-stage NWs could bend the NWs effectively to enhance the piezotronic effect and had a power output of about 5.9 mW·m^−2^ when used as a generator, which was 11 times higher than that of the planar type (~0.5 mW/m^2^), as shown in Figure 2e. Moreover, the layered NW in the piezoelectric mode exhibited excellent mechanical durability under repeated compression cycles (>1000 cycles). In addition to the multilayer structure, textile fiber type devices, such as ZnO and SiO_2_ woven into mixed fibers, and flexible devices made into thin films show excellent sensitivity and durability [103].

In the field of gas sensors, Wei et al. demonstrated highly sensitive gas sensors with Schottky contacts using one-dimensional (1D) ZnO NW structures [104]. The Ohmic contact device and Schottky contact device were prepared by fixing the two ends of NW on the Pt electrode and controlling the contact type of the two ends of NW. The result of the two sensors is shown in Figure 2f. The Schottky contact gas sensor could detect CO at only 50 ppm, and the sensitivity was improved by four orders of magnitude compared to the Ohmic contact device. Zhou et al. reported a room-temperature gas sensor with a metal–semiconductor–metal (M-S-M) structure [105]. ZnO NW was prepared using the VLS method and transferred to a flexible substrate; the *c*-axis was parallel to the substrate, and the sensitivity of the device was controlled by controlling the deformation of the NW, changing the Schottky barrier of the device, as shown in Figure 2g. The test output current for H_2_ increased by 5359% and NO_2_ by 238.8%, as shown in Figure 2h.

The fabrication process of molecular sensors is similar to gas sensors and mostly involves using Schottky contact one-dimensional ZnO NW devices [106]. ZnO NW is prepared by the VLS method and transferred to a flexible substrate, followed by modification of NWs according to different target molecules—generally, a solution for soaking the corresponding functionalized molecules. For example, to detect cDNA, the NWs are soaked in ssDNA solution, and the cDNA will bind to ssDNA and adsorb on the surface of the NWs, and when a compressive strain of −0.59% is applied to it, the relative current response is increased by 454%, as shown in Figure 2i [107]. The detection of other molecules, such as proteins [100] and proteoenzymes [36,108], is similar.

With the development of healthcare and improvement in the awareness of health detection, electronic devices driven by biological signals have attracted extensive attention. Therefore, biosensors have been developed rapidly. As an important nutrient that the human body can directly absorb, glucose plays an important role in biological activities. Therefore, the detection of glucose in the human body is very important. Wang et al. fabricated a glucose sensor driven by a triboelectrical nanogenerator based on the metal–semiconductor–metal structure of a single ZnO NW. Using the piezotronic effect to modulate the performance of the device, the sensing resolution and sensitivity as the key parameters of sensors were enhanced by more than 200% and 300%, respectively, and the output signal was improved by more than 200% under a compressive strain of −0.67%. This self-powered glucose sensor has great potential for development in the biomedical field [90]. Based on these studies, Wang et al. realized the reversible conversion of Schottky contact and Ohmic contact by utilizing a triboelectrical nanogenerator to tune the Schottky barrier height. High-sensitivity biosensors based on the reversible conversion between Schottky contact and Ohmic contact can realize the multifunctional detection of neurotransmitters and neuronal electrical signals. At present, the detection of dopamine and the recording of neural electric impulses have been realized. This reversible adjustment method of Schottky contact provides a new method for expanding ZnO NW devices [84]. In addition, as the important genetic material of the human body, the detection of DNA has been a necessary item for many medical institutions. Therefore, developing a simple, low-cost, and high-sensitivity detection method has attracted much attention. Wang et al. prepared a label-free DNA sensor based on a single ZnO NW device with Schottky contact. By introducing a compressive strain of −0.59%, the relative current response of the sensor in detecting the human immunodeficiency virus 1 gene was improved by 454% [107]. Meanwhile, Wang et al. also demonstrated the higher sensitivity of ZnO NW sensors with Schottky contacts compared with nanosensors with Ohmic contact because Schottky contact can clearly distinguish the polarity of piezoelectric charges [86,87]. This performance-tunable label-free detection method has great potential in the biomedical field.

### 3.3. Piezotronic Effect in Strain-Gated Transistors

A piezoelectric transistor modulates the transmission characteristics of the device by controlling the Schottky barrier [52]. Early studies of the piezoelectric properties of a single ZnO NW [10,109] and piezoelectric transistors [51] provide insight into the physical and electrical properties of ZnO NWs.

**Figure 3:**
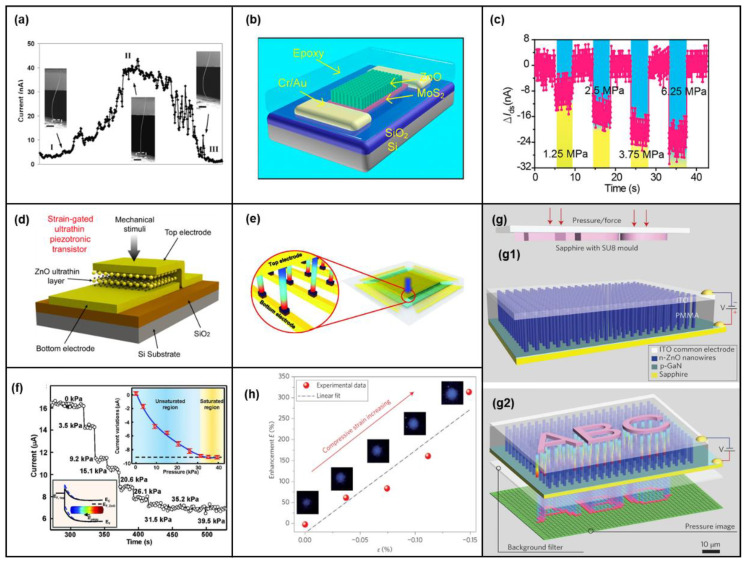
(**a**) The current value of the voltage-controlled NW transistor under the action of different forces. Reproduced with permission from [42]. Copyright (2006) *Nano Letters*. Structure and piezoelectric characteristics of pressure-gated field-effect transistors based on 2D MoS_2._ (**b**) Device structure. (**c**) Change of drain current at different pressures. Reproduced with permission from [115]. Copyright (2016) *American Chemical Society Nano*. (**d**) Schematic diagram of an ultra-thin transistor with a channel length of 2 nm. Reproduced with permission from [6]. Copyright (2018) *American Chemical Society Nano*. The structure diagram of the ZnO NW-based tactile sensing array is the current characteristic diagram. (**e**) A 3D piezo-gated transistor array schematic diagram and the individual device block diagram (enlarged schematic). (**f**) The current of a single tactile probe at different pressures corresponds to the current. Reproduced with permission from [119]. Copyright (2013) *Science*. Piezoelectric nanowire LED array structure diagram with pressure response of enhancement factor. (**g**,**g1**,**g2**) are sensor arrays before and after strain is applied, respectively. (**h**) Pressure-response diagram of the LED enhancement factor and the corresponding emitted light image. Reproduced with permission from [37]. Copyright (2013) *Nature Photonics*.

Wang et al. proposed a piezoelectric field effect transistor [42], as shown in Figure 3a, that can control the current of ZnO nanowires by generating an electric field through the bending of the nanowires. This piezoelectric field is the gate of the transistor. Peng et al. presented a field-effect transistor based on a free-standing piezoelectric fine wire (PFW) [110]. The ZnO portion buried in the PDMS root in the device is a key functional area, and the device has micron-level control sensitivity. Zhou et al. proposed to control the Schottky barrier height using the piezoelectric potential to realize the change of the I-V characteristics of NW devices from symmetric to diode-type [111]. Wang et al. demonstrated the use of vertically arranged ZnO NW strain-gated piezoelectric transistors to investigate the role of piezotronic effect modulation devices under different strains [112]. Xu et al. manipulated individual ZnO NW using the tip of a scanning tunneling microscope and found that when a bending strain of 2.63% was applied, the conductivity of the NWs decreased by two orders of magnitude [113]. For safe and simple use of the piezotronic effect, ion gel was precisely prepared on ZnO NWs. Applying a negative gate voltage through ion gel gating also improves the strain factor of the piezoelectric sensor and the output characteristics of the piezoelectric inverter [114]. Liu et al. prepared the first hybrid gated transistor based on carbon nanotubes and ZnO NWs to control charge transport in nanotubes through the piezoelectric potential of ZnO NWs [9]. Taking advantage of special material advantages, two-dimensional MoS_2_ was combined with one-dimensional ZnO NWs [115], as shown in Figure 3b. Figure 3c shows the change in drain current at different pressures with a drain voltage of 0.5 V and a gate voltage of 0 V. When a pressure of 6.25 MPa was applied across the packaged device, the source leakage current could be regulated by ~25%, which is equivalent to applying a back gate voltage of −5 V. For field-effect transistors, the control force of strain gating is very important, and only 806 nN is required in a single In-doped ZnO nanoribbon strain gated piezoelectric transistor to control the transistor switch, and the switching current ratio is 743 [66].

In addition to NW transistors, there are many piezoelectric transistors of other structures [116]. Wang et al. reported ultra-thin transistors with a channel length of only 2 nm [6], as shown in Figure 3d. The ultra-thin ZnO film introduces an extremely high electric field after applying stress, and only mechanical stress guidance is required to generate the gate signal itself. Wang also studied the influence of the spatial distribution of the piezoelectric potential on ZnO transport characteristics [117]. Different contact locations on the wire were selected to form metal-ZnO contacts. When the ZnO was in a non-uniform deformation state, the local piezoelectric potential distribution at different positions showed a significant influence and obvious change trend on the charge carrier transport characteristics. Wang et al. further studied the effect of the piezoelectric potential in ZnO NWs on the transport characteristics of NW field-effect transistors under different strain conditions, such as tensile, compression, and twisting [11]. Liu et al. also used first-principles calculations to obtain the width of the piezoelectric charge distribution in the Ag-ZnO-Ag transistor, which is a key parameter for understanding the piezoelectric effect [118]. Schottky barrier modulation at the interface of the two transistors exhibited asymmetric behavior due to the piezoelectric effect, which was consistent with previous experimental results. Wu et al. studied the current modulation and piezoelectric response of the dual-gate ZnO thin-film transistor (TFT) through experiments and analysis [38]. The analytical model reproduced the experimental piezoelectric response of the two-gate TFT well.

Furthermore, Wu et al. designed integrated arrays of piezoelectric transistors based on vertical ZnO NWs [119], as shown in Figure 3e. The pressure sensitivity of a single 3D strain-gated vertical piezotronic transistor (SGVPT) is shown in Figure 3f. The device exhibited high sensitivity under pressures below 10 KPa and could distinguish a maximum pressure of about 30 KPa. Independent addressing could be achieved, and mechanical stimuli applied to the device could be converted into local electronic control signals. Liu et al. similarly designed a set of piezoelectric transistors that could be used for tactile imaging [39]. By assembling ZnO nanosheets into ordered nanosheet arrays, 2D piezotronic transistor arrays with an ultra-high spatial resolution with a maximum pressure/strain sensitivity of 60.97–78.23 meV/MPa were developed, much higher than other reported piezoelectric transistors and sensors reported before 2017. Song et al. demonstrated an array of piezoelectric transistors based on Li-doped ZnO films [4]. It could also be used for tactile imaging, and in their study, the piezoelectric effect of the sensing element was studied and qualitatively characterized. The strain sensitivity (strain coefficient) of the unit was as high as 199. Pan et al. fabricated a pressure-sensor array based on NW-based LEDs, as illustrated in Figure 3g [37]. The entire array had a pixel density of 6350 dpi. Each pixel consisted of a single n-ZnO NW/p-GaN light-emitting diode whose emission intensity depended on the strength of the strain-induced piezoelectric potential. Figure 3h shows the luminescence intensity of the device under different strains. The luminescence of the LEDs increased with strain, and the spatial resolution of the LED array device was up to 2.7 μm. Moreover, strain gauge gated transistors can not only be used as logic devices for simple operations [5] but also as fast storage devices for writing or erasing data [120,121].

### 3.4. The Piezotronic Effect in Biochemistry

**Figure 4:**
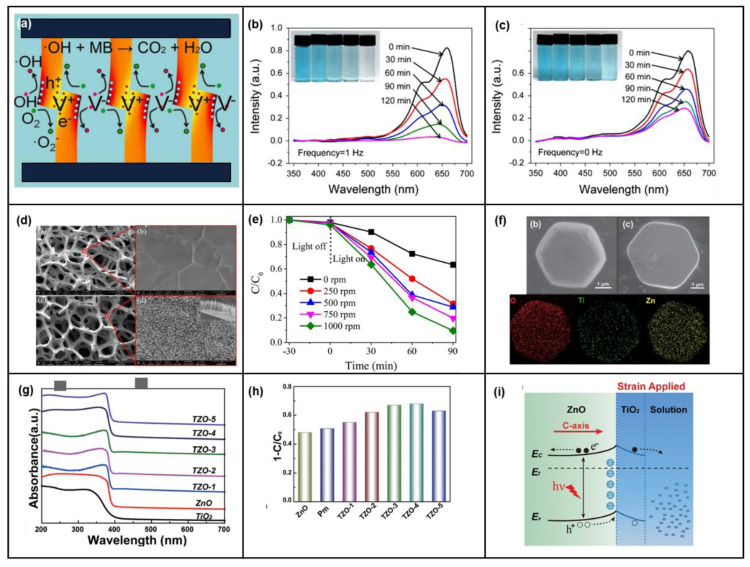
Schematic diagram of piezoelectric enhanced photocatalytic ZnO NW device and UV-Vis absorption spectroscopy. (**a**) Working mechanism of piezoelectric photocatalytic activity of ZnO nanowires. (**b**,**c**) UV-Vis absorption spectra after piezoelectric-assisted and non-piezoelectric-assisted photodegradation under different ultraviolet illumination times, respectively. Reproduced with permission from [54]. Copyright (2015) *Nano Energy*. SEM image of ZnO nanorod arrays/3D Ni foam and photocatalytic degradation of RhB on ZnO nanorod arrays/Ni foam. (**d**) On the left are Ni foam and ZnO nanorod arrays/Ni foam. The inset is the side-view SEM image. (**e**) Photocatalytic degradation yield of RhB on ZnO nanorod arrays/Ni foam at different stirring rates under UV light irradiation. Reproduced with permission from [122]. Copyright (2017) *Materials Today*. Structure, characteristics, and mechanism of the ZnO/TiO_2_ hybrid photocatalysts. (**f**) SEM images of a single ZnO/TiO_2_ hybrid sample and the corresponding mapping results. (**g**) Diffused reflectance UV spectra of the as-synthesized samples collected in the absorbance mode. (**h**) Degradation efficiency of MO in the presence of corresponding samples for 30 min. (**i**) The working mechanism of ZnO/TiO_2_ hybrid photocatalysts. Reproduced with permission from [123]. Copyright (2016) *American Chemical Society Nano*.

Environmental pollution is one of the most serious problems challenging the sustainable development of human civilization. Among them, in terms of the sewage-treatment scheme, purifying water based on the photocatalytic characteristics of solar energy is one of the most promising research directions. ZnO nanostructures have been thoroughly studied due to their high photocatalytic efficiency, low cost, and environmental friendliness. Xue et al. proposed a piezoelectric photocatalytic mechanism based on the electrostatic field and photocatalytic characteristics generated under the ZnO piezoelectric effect [54]. As shown in Figure 4a, ZnO NWs are hydrothermally grown on carbon fibers (CFs), and multiple CFs are woven together. Methylene blue (MB) is degraded by the piezoelectric photocatalytic activity of ZnO NWs/CFs in an aqueous solution with applied ultraviolet irradiation and periodically applied force. When ZnO NW is irradiated with ultraviolet light, the electrons are excited to transition to the conduction band, creating the same number of holes in the valence band. A piezoelectric field is generated on the surface by applying stress. The red area is compressive strain, which is negative piezoelectric potential, and the yellow area is tensile strain, which is positive piezoelectric potential. Under the action of the electric field, the photogenerated electrons drift to the surface of the positive piezoelectric potential, and the holes drift to the surface of the negative piezoelectric potential. Carriers’ drift due to the piezoelectric potential reduces the rate of recombination of photogenerated electrons and holes, and hence more carriers can drift to the surface.

The photogenerated holes on the negative electrode surface then react with hydroxyl groups to produce free OH radicals, and photogenerated electrons on the positive electrode surface react with O to produce O^2−^, as shown in Figure 4a. The comparison of Figure 4b,c shows that after 120 min, the degradation of MB in the solution with a vibration frequency of 1 Hz is as high as 96%, and that in the solution without piezoelectric assistance is only 64%.

To make more efficient use of the spatial configuration, Chen et al. reported an array of ZnO NWs on a three-dimensional Ni foam substrate and photocatalyzed organic pollutants in wastewater using piezoelectricity [122]. The electron microscopy diagram is shown in Figure 4d. The structural mechanism is like the previous device, and the water vortex generated by the three-dimensional structure causes ZnO NWs to sway, thereby generating a piezoelectric field on the surface and inhibiting the recombination of photogenerated carriers. The surface charge can then participate in the degradation reaction. Figure 4e shows the rate of photocatalytic degradation at different stirring rates. The faster the stirring rate, the stronger the piezoelectric field, and the faster the decomposition rate. In addition to applying additional force to ZnO NWs, it is also possible to modulate the strain in the semiconductor by direct sintering. Wang et al. demonstrated a hybrid photocatalyst with anisotropic strain inside by assembling TiO_2_ nanoparticles on ZnO monocrystalline nanoplatelets [123]. First, ZnO and TiO_2_ mixed samples were synthesized by the co-precipitation method. The electron microscopy and element distribution are shown in Figure 4f. The samples were then sintered at 450 °C for 2 h, removed, and cooled to room temperature in air, ice, and liquid nitrogen, named TZO-2, TZO-3, and TZO-4, respectively. Another sample was taken, further sintered at 200 °C, and naturally cooled to room temperature to relieve stress, named TZO-5. TZO-1 is a single ZnO chip. The XRD and Raman spectra of the samples were measured, and it was confirmed that the samples were subjected to varying degrees of compression strain along the *c*-axis. Its absorption spectrum was tested, as shown in Figure 4g, which showed a significant shift in the absorption edge of the sample. Figure 4h shows the decomposition rate under different strains, and it can be seen that TZO-4, which cooled the most violently, has the highest decomposition rate, which is caused by the highest internal strain. The piezoelectric potential and surface pressure polarization charge introduced by thermal stress change the band structure at the interface of ZnO and TiO_2_, promote the generation and separation of photogenerated carriers at the interface, and increase the photocatalytic degradation rate, as shown in Figure 4i. These studies show that the piezotronic effect has great potential in the field of biochemistry.

### 3.5. The Piezotronic Effect in Energy Harvesting

Energy has always been a global focus. Due to global warming, research on sustainable energy has been increasing. As some of the most abundant energy sources in nature, the harvesting and utilization of solar and mechanical energy have attracted extensive research interest. Photoelectric conversion mainly depends on the p-n junction, organic thin film, or heterojunction. Mechanical energy conversion mainly relies on the piezotronic effect [124]. ZnO-based heterojunctions have attracted significant attention due to their simultaneous photoelectric conversion and piezotronic effects. It has been proved that piezotronics can adjust the height of the Schottky barrier through the piezoelectric potential to enhance the power output of a ZnO photocell and can also be used to realize high-output nanogenerators [55,56,57,59,125,126]. This also illustrates the potential of ZnO-based electronic devices for harvesting mechanical and optical energy [59,127,128,129,130]. The structure of ZnO NWs with the introduction of solid electrolyte and metal coating hybrid cells has been demonstrated to harvest both solar and mechanical energy using a dye-sensitized solar cell and piezoelectric nanogenerator. Under light illumination of a simulated sun emission (100 mW/cm^2^), the optimum power was enhanced by 6% after incorporating the contribution of the nanogenerator [131]. By integrating the nanogenerators based on a polarized poly (vinylidene fluoride) (PVDF) film and the solar cells with ZnO-poly(3-hexylthiophene) (P3HT) heterojunction, a hybrid energy cell was fabricated to simultaneously harvest solar, thermal, and mechanical energies [58]. Wang et al. further demonstrated that the current of the hybrid cell is controlled from an alternating current type to direct-current-like type by mechanical straining processes both in a dark condition and under light illumination [132]. Lattice strain on the output voltage of nanogenerators was theoretically proved by doping ZnO-based nanofilms with different elements, which also proved the modulation of the piezotronic effect in lattices from a microscopic point of view [133].

### 3.6. Piezotronic Effect in Other Fields

In addition to sensors, energy harvesting, and logic operations, experiments have demonstrated the regulation of the electrical properties of ZnO devices with different morphologies, such as flexible ZnO films, ZnO NWs, and ZnO nanobelts [134,135,136].

In the emerging field of spintronics, a key concept is the voltage-gate control of spin precession via the effective magnetic field generated by the Rashba spin–orbit coupling (SOC). However, the external power acts as the traditional gate voltage. This has a great influence on the stability of the device. Based on the piezotronic effect of ZnO/P3HT and MAPbI_3_/ZnO heterostructures, Wang et al. used the piezoelectric potential via external strain to control the spin photocurrent, which reduced the noise floor and significantly improved the stability [137,138]. Moreover, piezotronic effects can also affect the output of the laser. Whispering-gallery mode and single-mode lasing in ZnO microwire can also be adjusted by the piezotronic effect for high-precision photo-sensing process [139,140].

Furthermore, channel modulation and interface modulation can enhance other physics effects. The current of the as-constructed ZnO/SiC heterojunction NWs is 6 times greater than SiC NWs, indicating that the piezotronic effect enhances the sensitivity of the piezoresistive effect [141]. The gauge factor can be significantly enhanced by applying pressure to the surface of the 2D ZnO ultrathin nanosheet [142]. The interface property, such as surface potential and Schottky barrier height of ZnO microwire, can also be changed to affect the transport characteristics of carriers under UV laser illumination by coupling piezotronics and photoexcitation [143,144]. The tunneling effect can also be altered. Simulations by Zheng et al. revealed that external strain can effectively tune the transport properties of ZnO piezoelectric tunnel junctions, and the size dependence of the strain tunability exhibits a simple exponential law [145].

## 4. Piezo-Phototronics Based on ZnO Nanowires

The piezo-phototronic effect is the three-field coupling effect of piezoelectricity, photoexcitation, and a semiconductor. For non-centrosymmetric semiconductor materials, we can use the piezoelectric potential to adjust and control the generation, separation, and transport of carriers in the interface and junction regions, as well as other recombination processes. Novel high-performance optoelectronic devices can be realized through the research of piezo-phototronics, which have been widely used in various fields [146,147]. In this section, the theoretical research on the piezo-phototronic effect and the research progress in photodetectors, photodiodes, solar cells, and other fields are summarized by analyzing the different structural systems formed by ZnO and other materials [148,149].

### 4.1. Theoretical Research of the Piezo-Phototronic Effect

Both ZnO thin films and ZnO NW have piezoelectric properties, and both can be used for the study of the piezo-phototronic effect, but current research basically focuses on ZnO NW devices [150,151]. The main reason for this is that ZnO NWs can withstand large mechanical strains without breaking compared with ZnO thin films and can also be prepared into desired shapes using photolithography and low-temperature hydrothermal methods. The preparation process is simple and low-cost, and the response to mechanical strain is higher [8,152]. Thus, most studies have focused on ZnO NW devices. At the same time, current theoretical studies have demonstrated that the piezoelectric potential introduced by mechanical strain changes the Schottky barrier height and the energy band of the p-n junction formed between n-ZnO NWs and metals or p-type semiconductors/polymers [40,153,154]. Therefore, the piezo-phototronic effect can enhance the performance of optoelectronic devices such as photodiodes [155,156], LEDs [156], photodetectors [157], and solar cells [1,158] by tuning the barrier height and depletion region width at the interface.

### 4.2. The Piezo-Phototronic Effect in Photodetectors

**Figure 5:**
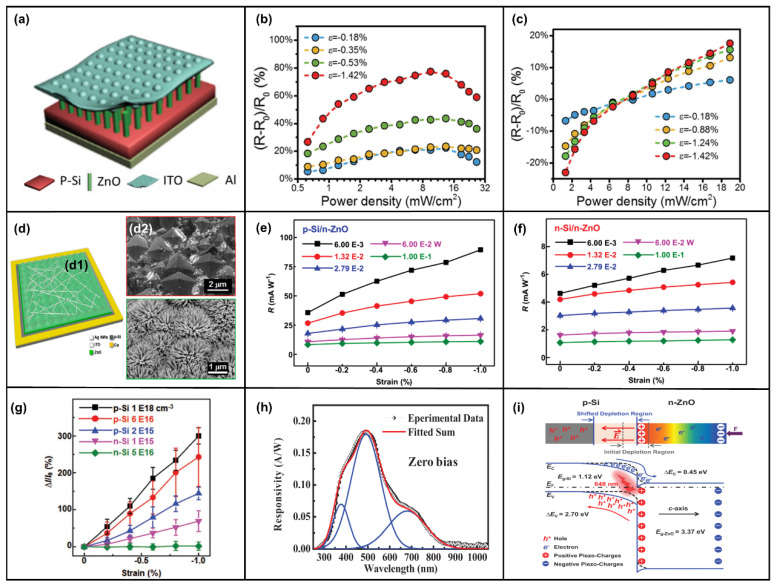
Device structure and characterization of Si/ZnO photodetector. (**a**) Device structure of p-Si/n-ZnO NWs photodetectors. (**b**,**c**) Relative changes of photoresponsivity of the device under different strains and illumination of 442 nm and 1060 nm at −2 V, respectively. Reproduced with permission from [159]. Copyright (2017) *Advanced Materials*. (**d**) Schematic structure of a p-Si/n-ZnO NW hybridized photodetector etched by KOH. Reproduced with permission from [160]. Copyright (2014) *American Chemistry Society Nano*. (**e**,**f**) The photoresponsivity as a function of compressive strain under different optical powers for the p-Si/n-ZnO NWs (at −1 V) and the n-Si/n-ZnO NWs (at 1 V) heterojunction photodetectors. Reproduced with permission from [161]. Copyright (2018) *Advanced Functional Materials*. (**g**) The average relative changes in photocurrent as a function of external compressive strain under 1.90×10^−3^ W laser power for the Si/ZnO heterojunction photodetectors with different doping types and concentrations of Si. Reproduced with permission from [162]. Copyright (2020) *Advanced Functional Materials*. (**h**) Deconvoluted spectral responsivity at zero bias (0 V). Reproduced with permission from [164]. Copyright (2017) *Journal of Physics D-Applied Physics.* (**i**) Schematic energy band diagrams of the p-Si/n-ZnO NWs without (dashed black line) and with (solid blue line) external compressive strain to 648 nm laser illumination. Reproduced with permission from [162]. Copyright (2020) *Advanced Functional Materials*.

ZnO is a wide-bandgap semiconductor. Most of the current photodetectors based on ZnO NWs are ultraviolet photodetectors. By changing the material and device structure, the detection of ultraviolet light with different wavelengths can be realized. The basic photodetectors use ZnO NWs and different p-type semiconductors/polymers to form heterogeneous p-n junctions. Photodetection is realized by utilizing the absorption and separation ability of the depletion region for the photogenerated carriers. Then, the piezo-phototronic effect is used to adjust the barrier height of the junction region and the width of the depletion region to improve the responsivity of photodetection. Silicon-based p-n junction photodetectors play an important role in optoelectronic applications due to their excellent compatibility with well-developed integrated circuit technologies [159]. As shown in Figure 5a, n-ZnO NWs are grown on p-Si as a substrate to form a heterojunction. Si can absorb light with a longer wavelength due to a narrower forbidden bandwidth. ZnO can absorb light with a shorter wavelength as a wide-bandgap semiconductor. Thus, broadband light detection from visible light to ultraviolet light is realized [159]. The study in Figure 5b also shows that the responsivity to 442 nm light increased by 78% after introducing the piezo-phototronic effect. In Figure 5c, although the responsivity of 1060 nm light was only increased by 18%, there was no saturation, and it had good linearity, which also shows the detection ability of near-infrared light [159]. In Figure 5d, the Si substrate was etched with KOH into a pyramid structure with a larger light-receiving area. The performance improvement was significant, including a 177% increase in responsivity and an 87% reduction in response time for 442 nm UV-visible light [160]. In addition, Peng et al. compared the modulation of the piezo-phototronic effect in photodetectors based on n-Si/n-ZnO and p-Si/n-ZnO heterojunctions with different doping concentrations [161,162]. The research results of Figure 5e,f show that the responsivity improvement of the inversion heterojunction was much greater than that of the homotype heterojunction for 405 nm light [161]. Figure 5g also shows a better current response to 648 nm light at a high doping concentration of 1 × 10^18^ cm^−2^ with different p-Si dopings [162]. Hu et al. also demonstrated that when a compressive strain of 0.7 N was applied, the response characteristics of UV light and visible light were improved by 67.3% and 74.5% at high doping concentrations compared with low doping concentrations [163]. In addition, replacing ZnO NWs with flexible ZnO films causes responsivity to reach 0.20A/W for 490 nm light, as sketched in Figure 5h [164]. The simulation results of COMSOL Multiphysics software also theoretically proved that the piezo-phototronic effect modulates the flexible ZnO thin film’s response to 405 nm light [165]. Generally speaking, the main method adopted by the piezo-phototronic effect for p-Si/n-ZnO heterojunction modulation is to introduce positive piezo-charges on the ZnO interface side to repel electrons and increase the width of the depletion region. The schematic energy band diagram of the p-Si/n-ZnO NWs is shown in Figure 5i. The responsivity was improved by increasing the generation rate of photogenerated electron–hole pairs.

**Figure 6:**
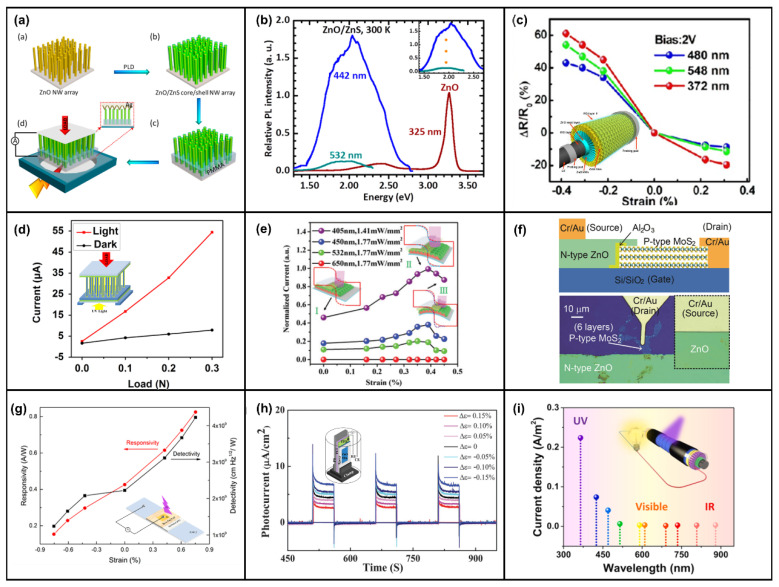
Device structure and characterization of photodetectors formed by Si/organic or other inorganic materials. (**a**) Schematic illustration of the ZnO/ZnS CS NW photodetector fabrication process. (**b**) Room-temperature PL for ZnO/ZnS core/shell NWs collected with comparable laser power ∼145 μW under 532, 442, and 325 nm excitation, respectively. Inset: Magnification of the type-II transition PL peaks. The dotted orange line marks the broad peak center at ∼1.95 eV. Reproduced with permission from [166]. Copyright (2015) *American Chemistry Society Nano*. (**c**) Relative changes of responsivity. Inset: Schematic of a single carbon-fiber/ZnO-CdS wire-based photodetector. Reproduced with permission from [171]. Copyright (2013) *American Chemistry Society Nano*. (**d**) The plots of current at a fixed bias of 3.5 V as a function of pressure with/without illumination. Inset: Schematic diagram of the pressure sensor. Reproduced with permission from [175]. Copyright (2016) *Nano Energy*. (**e**) Photocurrent of the photodetector with different strains under 405 nm (1.41 mW/mm) and 450, 532, and 650 nm (1.77 mW/mm) illumination. Reproduced with permission from [178]. Copyright (2019) *Nanoscale*. (**f**) Side view of the schematic structure of the p-MoS2 and n-ZnO diode (top) and optical image of a typical device (bottom). Reproduced with permission from [189]. Copyright (2016) *Advanced Materials*. (**g**) Curves of calculated responsivity and detectivity as a function of applied strains, respectively. Inset: Schematic diagram of the device structure. Reproduced with permission from [192]. Copyright (2016) *American Chemistry Society Applied Materials & Interfaces*. (**h**) Photocurrent of the device under varying compressive and tensile strains at zero bias. Inset: A schematic of the device. Reproduced with permission from [194]. Copyright (2014) *Physical Chemistry Chemical Physics*. (**i**) Current densities of the photodetector under light illuminations with different wavelengths. Reproduced with permission from [195]. Copyright (2022) *Nano Energy*.

In addition to heterojunction formation with Si, ZnO has been studied with other p-type materials. Meanwhile, ZnO/ZnS and ZnO/CdS type-II heterojunction core–shell (CS) NW arrays have been fabricated. The CS NW array formed by ZnO and ZnS shown in Figure 6a not only has high responsivity to blue light, green light, and UV light but can also detect photons with an energy of 2.2 eV (less than the ZnO and ZnS bandgap), which is mainly attributed to spatially indirect type-II transition facilitated by the abrupt interface between the ZnO core and ZnS shell, as shown in Figure 6b [166]. Moreover, a wurtzite (WZ) ZnO/WZ ZnS CS NWs and WZ ZnO/zinc blend (ZB) ZnS CS NWs were also fabricated, and the results showed that the former had greater electrical transport and phot-sensing properties under external strain [167]. The ZnO/ZnS CS NWs with a horizontal structure still had a responsivity of 1.19×10^2^ A/W, which was 23.9 times that of the ZnO NW structure [168]. CdS and ZnO could form a core–shell structure. In addition to having a high response to 548 nm green light and 372 nm UV light [169], it could also form a mixed structure with optical fiber [170,171]. As shown in Figure 6c, the photodetector exhibited ultrahigh photon responsivity under the illumination of blue light (1.11 × 10^5^ A/W, 480 nm), green light (3.83 × 10^4^ A/W, 548 nm), and UV light (1.94 × 10^5^ A/W, 372 nm), respectively. Additionally, the photoresponsivity increased by 60% at a compressive strain of 0.38% [171]. ZnO/NiO CS NWs were similar to the ZnO/ZnS structure, and the visible light with smaller photon energies (3.0 eV) than the band gap of ZnO (3.3 eV) and NiO (3.7 eV) could be sensitively detected due to the spatially indirect type-II transition between ZnO NWs and NiO film, and the enhancement in photocurrent and sensitivity of the UV photodetector was about 74% and 78.7% under a 1 N compressive strain at 3.5 V, respectively [172,173]. A ZnO/ZnSe heterostructure of the same type could also have high responsivity to external UV light and visible light [174]. The performance of ZnO/NiO CS NWs pressure sensors can also be enhanced by the piezo-phototronic effect by reducing the bulk resistance (effective separation of photogenerated electron–hole pairs increases the free-carrier concentration), as shown in Figure 6d [175]. The piezo-phototronic effect can enhance the photodetection performance of the p-Cu_2_O/n-ZnO NW heterojunction by 18.6% at a compressive strain of 0.88% [176], and embedding the 405 nm LED can also increase the linear dependence of the switch ratio of the Cu_2_O/ZnO NW-based pressure sensor by 376% [177]. In addition, the photoresponse of the serrate-structured Cu_2_O substrate is 2.5 times that of the planar structure, and the performance is significantly improved by 5 times when a compressive strain of 0.39% is applied, as shown in Figure 6e [178]. The UV responsivity of the p-CuI-ZnO film/NW heterojunction can be enhanced by the piezo-phototronic effect [179,180]. The responsivity of ZnO and Mg-doped MgZnO (MZO) and Al-doped AlZnO (AZO) thin films to UV light was also significantly improved after pressure was applied [181,182]. The responsivity of the doped structure formed by AZO and MZO was enhanced by 306% and 210% under specific deformation and ultraviolet light of 320 and 345 nm, respectively [183]. Of course, in addition to these common materials, the piezo-phototronic effect can also modulate the p-n junction formed by n-ZnO and other materials. A 3C–SiC/ZnO NW heterostructure can achieve a high responsivity of 1.01 × 10^6^ A/W and a low response time of 24.1 ms at a compressive strain of 0.381% [184]. The photocurrent of the SnO nanosheet/ZnO NW structure increases by 55 times under the pressure of 2 mN relative to the free strain [185]. The photocurrent of the ZnO/Ga_2_O_3_ heterojunction triples at 0.042% compressive strain to 261 nm deep ultraviolet light [186]. The GaN/ZnO heterostructure has an outstanding detection sensitivity of 6.82 × 10^13^ Jones for 325 nm ultraviolet light under zero bias [187]. It is worth mentioning that the photoresponsitivity and detectivity of the photodetector based on 2D ZnO nanosheets/GaN nanorods were enhanced by two orders of magnitude compared to 1D GaN nanorods [188]. The 2D p-MoS_2_/n-ZnO diode not only has a high rectification ratio (3.4 × 10^4^) but also has the highest external quantum efficiency (EQE), which can reach 52.7% at −2 V, as shown in Figure 6f [189]. The graphene/ZnO NW mixed dimensional van der Waals heterostructure prepared on a flexible substrate can be used as a photosensor and pressure sensor with high responsivity and switching speed [190,191]. In addition to forming heterojunctions with compounds and elementary substances, ZnO NWs can also form heterojunctions with other organic p-type polymers for photodetection. In Figure 6g, the ZnO NWs/Spiro-MeOTAD heterojunction UV photodetector could be modulated by the piezo-phototronic effect. Together with responsivity and detectivity, the photocurrent could be increased about 1-fold upon applying a 0.753% tensile strain [192]. As shown in Figure 6h, the photocurrent of the heterojunction formed by ZnO NWs and organic electrolyte was significantly enhanced under strain modulation [193,194]. In Figure 6i, the photodetection based on ZnO/P3HT heterostructure could detect photons in the visible and near-infrared bands, and the photoresponsivity was increased by 81.2% under the modulation of the piezo-phototronic effect [195].

**Figure 7:**
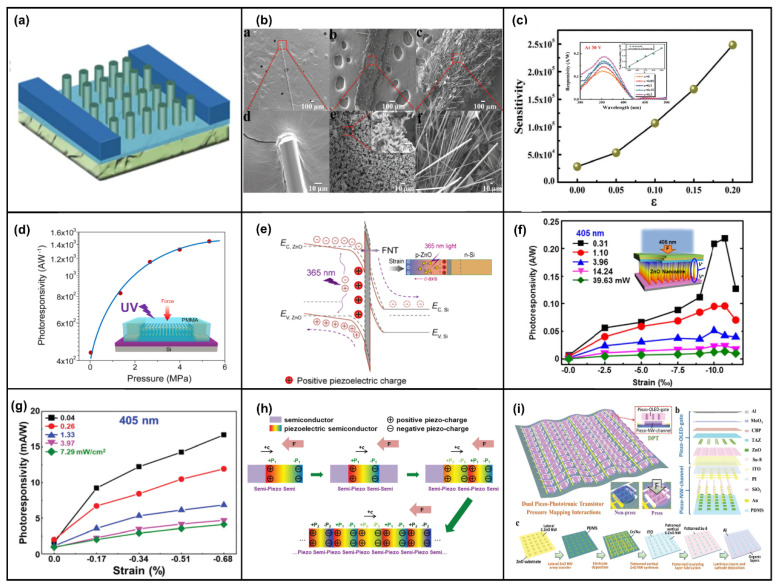
Device structure and characterization of the M-S-M and multilayer structure formed by ZnO NWs. (**a**) Schematic illustration of Ag–ZnO–Ag photodetector. Reproduced with permission from [197]. Copyright (2018) *Royal Society of Chemistry Advances*. (**b**) Field-emission scanning electron microscopy images of as-synthesized ZnO microarchitectures: ZnO microwire, ZnO coral-like microstrip, and ZnO fibril-like clustered microwire. Reproduced with permission from [199]. Copyright (2018) *Small*. (**c**) Sensitivity as a function of the tensile strain. Inset: responsivity spectra of the photodetector with different tensile strains under 30 V. Reproduced with permission from [200]. Copyright (2019) *Advanced Materials Interfaces*. (**d**) Photoresponsivity of ZnO photodetector as a function of applied pressure at M-S contact. Inset: device structure. Reproduced with permission from [203]. Copyright (2021) *Nano Energy*. (**e**) Schematic band diagrams of the piezo-phototronic effect in p-ZnO/Al_2_O_3_/n-Si junction. Reproduced with permission from [212]. Copyright (2021) *Nano Energy*. (**f**) The photoresponsivity at a bias of −2 V as a function of the compressive strain under different illuminant powers for 405 nm laser illuminations. Inset: 3D schematic device of the n-Si/n-ZnO/p-PEDOT: PSS tri-layer structure. Reproduced with permission from [30]. Copyright (2018) *Nano Energy*. (**g**) Photoresponsivity of the p-Si/i-ZnO/n-ZnO photodiode as a function of externally applied compressive strain. (**h**) Perspectives on piezo-charge engineering at multi-layer piezo-semi optoelectronic device interfaces. Reproduced with permission from [216]. Copyright (2020) *Journal of Materials Chemistry* C. (**i**) Schematic illustration and fabrication processes of the designed dual piezo-phototronic transistor pressure-mapping interaction system with the dual piezo-phototronic transistor array. Reproduced with permission from [217]. Copyright (2020) *Advanced Functional Materials*.

The second structure was based on the M-S-M structure formed by ZnO NWs and different metals. The piezo-phototronic effect was used to adjust the height and width of the Schottky barrier to improve the transport efficiency of carriers and improve the responsivity of photodetection [15]. Wang et al. demonstrated the enhancement of the photoresponsivity of M-S-M structures based on single ZnO NWs by piezo-phototronic effect [196]. In Figure 7a, ZnO NWs and Ag form a flexible M-NW-M structure with Schottky contact. The photocurrent and sensitivity of the UV photodetector are increased by 82% and 130% at 1 V at 0.2% tensile strain, respectively [197]. In addition, the M-S-M structure formed by ZnO nanosheets and Ag can also be effectively modulated by strain [198]. Sang-Jae Kim et al. also comparatively studied the modulation of the piezo-phototronic effect in the structures of ZnO microwire, ZnO coral-like microstrip (CMS), and ZnO fibril-like clustered microwire (F-MW), as shown in Figure 7b. The research showed that ZnO F-MW a higher photocurrent (I_Ph_) response (I_Ph/ZnO F-MW_ > I_Ph/ZnO CMS_ > I_Ph/ZnO MW_) [199]. The responsivity of the Au-ZnO thin film-Au structure photodetector was improved by only 20%, but the sensitivity was increased by 770% under the tensile strain of 0.2, as shown in Figure 7c [200]. Jiang et al. demonstrated the enhanced response speed of the Au-ZnO film-Au structure and the Au-AZO film-Au structure via the piezo-phototronic effect [201,202]. As shown in Figure 7d, an atomically thin ZnO field effect transistor with a high photoresponsivity of 300 A/W (V_ds_ = 2 V) was fabricated based on 2D-ZnO nanosheet, which was more than 10^3^ times higher than the performance of commercial UV photodetectors, indicating the effectiveness of the piezo-phototronic effect on the nanoscale [203]. Wang et al. prepared large-scale photodetectors based on a Au-ZnO NWs-Au structure. The strain-induced piezo-charges effectively enhanced the performances of the UV photodetector array by 700% in terms of photoresponsivity, 600% in terms of sensitivity, and 280% in terms of the detection limit [204]. Ravinder Dahiya et al. designed a kirigami-inspired honeycomb-patterned ZnO NWs with a photo/dark current ratio of 5 × 10^4^ and a fast recovery time of 100 ms. Furthermore, the stretchable photodetector was coupled with a flexible triboelectric nanogenerator to demonstrate a self-powered system for real-time UV radiation monitoring [205]. Moreover, the photocurrent of the Pt-ZnO NWs-Pt structure increased by 176% at 0.62% compressive strain [206]. A novel self-powered flexible vision electronic skin was realized based on a Ti-ZnO NWs-Ti structure [207]. A modulated UV photodetector was realized through surface/interface carrier-transport control based on a self-bending-assembled In-ZnO NWs-In structure [208].

The third structure uses ZnO and other materials to form a multilayer structure. The content of this section is more diverse [16]. The coupling of the piezo-phototronics effect and the tunneling effect was studied by adding an insulating layer between metal and ZnO. The sensitivity of Au–MgO–ZnO NW UV photodetectors can be effectively improved, but the sensitivity decreases when the pressure limit is exceeded due to the increase in dark current [209]. The responsivity and detectivity of the Pt/Al_2_O_3_/ZnO-based self-powered photodetector were increased by 2.77 times and 2.78 times under a compressive strain of 1.0%, respectively [210]. The responsivity and sensitivity of p-Si/Al_2_O_3_/n-ZnO were nearly one order of magnitude higher than that of a reference device of p-Si/n-ZnO NW arrays via the quantum mechanical Fowler–Nordheim tunneling mechanism, significantly higher than the commercial silicon photodiodes as well. Additionally, the piezo-phototronic effect had different adjustment effects on 442 nm and 1060 nm light [211]. Switching the doping types of ZnO and Si, the photoresponsivity of p-ZnO/Al_2_O_3_/n-Si photodetector increased almost linearly with the increase in strain for UV light, visible light, and near-infrared light under higher light density. The modulation of the tunneling effect by piezo-phototronics is shown in the energy band diagram of Figure 7e [212]. Moreover, the photodetector based on Si/ZnO/CdO three-component heterojunctions had a fast response time (shorter than 0.45 s) to the UV and visible illumination at 0 V bias [213]. The photoresponsivity and detectivity of PVK/ZnO nanorods/graphene heterostructure UV photodetector were enhanced by 440% and 132% under UV light illumination by introducing 1.093% compressive strain, respectively [214]. A Cu(In,Ga)Se_2_ (CIGS) multilayer heterojunction of glass/Mo/CIGS/CdS/ZnO NW/ITO was prepared as a PD exhibiting excellent performances with a wavelength ranging from 405 to 1064 nm, responsivity of 0.455 A/W, and a detectivity of 7.22 × 10^11^ Jones at zero bias by coupling pyroelectric and piezoelectric effects [215]. In Figure 7f, the n-Si/n-ZnO/p-PEDOT:PSS tri-layer heterojunction PD was fabricated and systematically investigated the piezo-phototronic effect in its performances simultaneously, utilizing both positive and negative piezo-charges [30]. Moreover, a method was proposed that introduced a p-Si/i-ZnO/n-ZnO photodiode with high performance, which can rationally design the polarity of piezo-charges at the interface to significantly improve photodiode performance via piezo-phototronic effects, as shown in Figure 7g,h. Additionally, the experimental results were verified by the finite element method [216]. Jiang et al. reported a novel dual piezo-photo-transistor, as shown in Figure 7i, based on a piezo invert-structured organic light-emitting diode as a gate control and piezo NWs array, and the results indicated that this device could detect a large pressure gradient within a wide output range for high-dynamic-range pressure-mapping interactions [217,218].

Moreover, the rectification characteristics and photoresponse performance of a heterojunction photodetector made from p-Si/V doped-ferroelectric-ZnO 2D nanosheets were significantly improved in a broad spectral range by introducing the ferroelectricity and the piezo-phototronic effect [219]. The same characteristics can also be observed in a ferroelectric p-type La-doped bismuth ferrite film/n-type ZnO NW array heterojunction [220]. Additionally, the responsivity and detectivity of a self-powered photodetector based on ZnO/CuO nanorods covered with Au nanoparticles were dramatically enhanced by introducing the piezo-phototronic effect, pyro-phototronic effect, and localized surface plasmon resonance together [221]. Furthermore, the photocurrent of a self-powered organic/inorganic PD based on PEDOT:PSS/ZnO NWs was dramatically enhanced by over 600% by combining the pyro-phototronic and piezo-phototronic effects together [222]. The magnetically induced current response was enhanced by at least 2 orders of magnitude based on ZnO/Co_3_O_4_ core/shell heterojunction NW arrays under UV light illumination due to the coupling effect of magnetic fields on the piezotronic and piezo-phototronic effect [223].

### 4.3. The Piezo-Phototronic Effect in a Light-Emitting Diode

**Figure 8:**
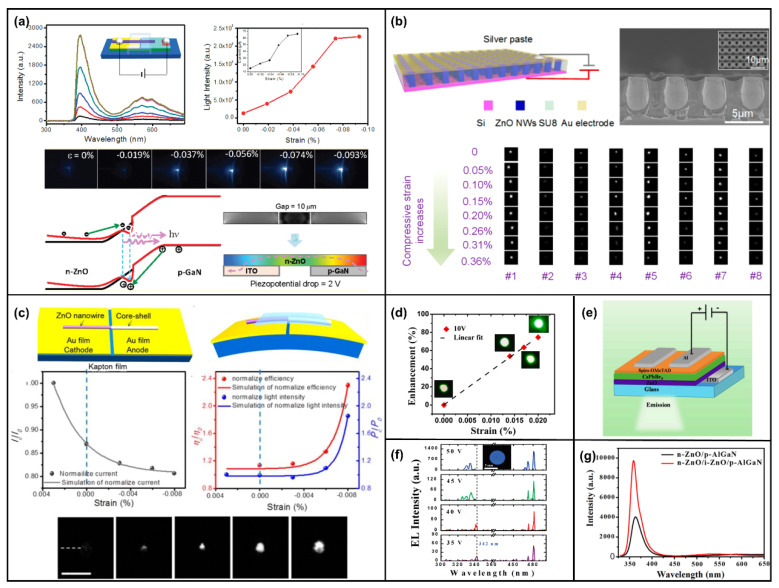
Enhancement in emission light intensity via the piezo-phototronic effect based on ZnO. (**a**) Enhancement in emission light intensity and band diagram of a n-ZnO NW/p-GaN LED under applied strain. Reproduced with permission from [226]. Copyright (2011) *Nano Letters*. (**b**) Device structure and enhancement in emission light intensity of ZnO-nanofilm/Si-micropillar heterostructure arrays. Reproduced with permission from [229]. Copyright (2016) *American Chemical Society Nano.* (**c**) Device structure and enhancement in emission light intensity of a ZnO NW/p-polymer CS UV LED. Reproduced with permission from [233]. Copyright (2013) *Nano Letters*. (**d**) The percentage enhancement in the light emission intensity of the fabricated LED under different applied strains up to ~0.02%. (**e**) Schematic representation of ITO/ZnO/CsPbBr_3_/Spiro-OMeTAD/Al heterojunction device configuration for EL measurements. Reproduced with permission from [237]. Copyright (2021) *Nano Energy*. (**f**) EL spectra recorded from the p-n ZnO NW arrays under various operating voltage conditions at room temperature. The top view photograph of the bright light-emitting spot at 50 V is displayed in the inset figure. The dashed vertical lines located at 342 nm were introduced as a guide for the eye. Reproduced with permission from [239]. Copyright (2010) *Nano Letters*. (**g**) The room-temperature PL spectra. Reproduced with permission from [238]. Copyright (2018) *Applied Physics Letters*.

In addition to adjusting the response performance of photodetectors, the piezo-phototronic effect can also enhance the luminous efficiency of light-emitting diodes (LEDs). This effect has been widely used in various device structures [224]. Since the lattice mismatch ratio between GaN and ZnO is low, it is thus an ideal p-type material to form a p-n junction with n-ZnO. Wang et al. demonstrated the capability of controlling the spatial distribution of the blue/near-UV LEDs composed of position-controlled arrays of n-ZnO NWs on a p-GaN thin film substrate by a conjunction of electron beam lithography and low-temperature wet chemical methods [225]. The piezo-potential acts as a “gate” voltage to tune the charge transport and enhance carrier injection by introducing the piezo-phototronic effect. The results indicated that the emission intensity and injection current were enhanced by a factor of 17 and 4 under 0.093% compressive strain, respectively [226]. The adjustment of pressure to light is shown in Figure 8a. The p-GaN/n-ZnO NWs heterostructure based on a flexible substrate with a high spatial resolution of 2.6 µm (much better than the human skin resolution of 50 µm) and a fast response time of 180 ms has potential for biomedicine science [3]. Additionally, the experimental results of adding MgO between p-GaN and n-ZnO NWs showed that the emission efficiency and injection current at a fixed applied voltage of 50 V were enhanced by a factor of 3.95 and 1.25 when introducing the piezo-phototronic effect, respectively [227]. Horizontally aligned ZnO microrod arrays with different orientations and periodic distributions can also be used as a building block for chip-in piezo-phototronic LEDs [228]. The light emission intensity of a Si/ZnO NWs heterostructure first increases and then decreases when the applied stress is increasing [229]. The results of modulations of the device structure and compressive strain are shown in Figure 8b. In addition, the n-ZnO nanofilm/p-Si micropillar heterostructure LED array was fabricated for light emissions by etching the Si [230]. Flexible LED can also be prepared by transferring the n-ZnO nanofilm/p-Si micropillar heterostructure LED array to a flexible substrate [231]. These results indicate a promising approach for fabricating Si-based light-emitting components with high performance enhanced by the piezo-phototronic effect [230,231]. The efficiency of the trap-assisted green electroluminescence was also enhanced by adding a SiO_2_ layer between ZnO NW and Si NW because the piezoelectric field built inside the component ZnO NW improved the recombination rate of the electron–hole pairs [232]. PEDOT: PSS/ZnO NW hybridized LEDs with very high light-emitting uniformity were also prepared, and the result indicated that the emission intensity could be enhanced by applying an external strain due to the piezo-phototronic effect, which reduced the barrier height for carrier transport, leading to an improved balance between the electron-contributed current and hole-contributed current [2,233]. The structure and CCD images recorded from the emitting end of a packaged single-wire LED under different applied strains are shown in Figure 8c. High emission intensity is still maintained on flexible substrates [234]. Light emission of a ZnO/TAZ heterostructure piezo-OLED is also enhanced by the piezo-phototronic effect and used for pressure sensors [235]. Furthermore, performances of white LED based on CsPbBr_3-x_I_x_(x = 0–3)/ZnO heterojunctions on a flexible platform and a high-resolution infrared LED array based on an ordered ZnO NW/SiGe heterojunction could also be improved by introducing strains [236,237]. The strain-modulation results of the piezo-phototronic effect and device structure of a ITO/ZnO/CsPbBr_3_/Spiro-OMeTAD/Al heterojunction are shown in Figure 8d,e. As shown in Figure 8f,g, a near UV LED made with in situ doped p-n homojunction ZnO NW arrays and n-ZnO/i-ZnO/p-AlGaN heterojunction film LED were fabricated, and their performances were still significantly enhanced by the piezo-phototronic effect [238,239].

In short, the main working mechanism of LED light emission is that the p-n junction releases the excess energy in the form of photons when the carrier is injected and recombined with an external bias. The piezo-phototronic effect can adjust the energy band structure at the interface to form a hole tap or electron trap. Therefore, the carrier injection efficiency is improved, and the performance is enhanced. This is an efficient method to increase the carrier recombination rate. Subsequently, the EQE of the LED and light emission intensity are enhanced.

### 4.4. The Piezo-Phototronic Effect in Solar Cells

The basic working principle of solar cells is the photovoltaic effect. When sunlight or other light irradiates the p-n junction of the semiconductor, electron–hole pairs are generated. The carriers generated near the p-n junction inside the semiconductor are not recombined and reach the space charge region. They are attracted by the internal electric field. Thus, the electrons flow into the n-region and the holes flow into the p-region. The n-region stores excess electrons, and the p-region has excess holes. They form a photogenerated electric field opposite the direction of the potential barrier near the p-n junction. In addition to partially offsetting the effect of the barrier electric field, the photogenerated electric field also makes the p-region positively charged and the n-region negatively charged. An electromotive force is generated in the thin layer between the n-region and the p-region. However, low conversion efficiency has been the main limiting factor for the solar cell industry. A large portion of the energy loss during solar-cell operation is attributed to optical loss, namely, the loss of the incoming light by reflection. In addition to the NW structure and micropyramid structure being able to significantly improve the conversion efficiency [240], the piezo-phototronic effect can also regulate the generation, transport, and recombination of carriers at the interface through the piezoelectric potential. Therefore, it can be used to adjust the conversion efficiency of solar cells. A silicon-based nanoheterostructure (p^+^-Si/p-Si/n^+^-Si and n-Si/n-ZnO NWs) device was fabricated, and the result demonstrates enhanced performance through significantly enhanced light absorption in the NW array and effective charge carrier separation by the piezo-phototronic effect [241]. Moreover, the power-conversion efficiencies of a flexible single ZnO NW/perovskite solar cell were improved from 0.0216% to 0.298% due to the piezo-phototronic effect [242]. Furthermore, the power-conversion efficiencies of a flexible ZnO NW array/perovskite solar cell were improved from 9.3 to 12.8% under a static mechanical strain of 1.88% [243]. The energy-conversion efficiency of the CS ZnO@ZnS NWs/perovskite solar cell was also significantly improved through the piezo-phototronic effect [244]. Flexible solar cells based on a n-ZnO/p-SnS CS NW array, a ZnO NW-based CIGS solar cell, and a ZnO/P3HT solar cell system with different crystallizations and doping levels of ZnO have all been studied, and the results show a significant increase in power-conversion efficiencies due to the piezo-phototronic effect [245,246,247].

### 4.5. The Piezo-Phototronic Effect in Other Fields 

In addition to modulating the performance of photodiodes, LEDs, and solar cells, the piezo-phototronic effect has a wide range of applications in other fields. Artificial synapses are critical information processing units in neuromorphic computing systems. Applying additional stimulation to the photonic synapse will lead to a multi-level adjustment of synaptic plasticity behavior, which is useful for developing a neuromorphic system with fast speed, large bandwidth, and high reliability. A transparent and flexible photonic synapse based on a single ZnO NW with modulation by the piezo-phototronic effect was fabricated, and the weight change as the key parameter characterizing the performance of artificial synapses was reduced from 1437.5% to 191.4% with a compressive strain change from −0.00% to −0.28% under 4.2 mW/cm^2^ UV light illumination [248]. The performance of a highly transparent and flexible two-terminal ZnO/Ag-NWs/PET photonic artificial synapse was also modulated by the piezo-phototronic effect, and the experimental results are quantitatively explained as a dynamic of photo-induced electron–hole trapping/detraining via defect states, such as oxygen vacancies [249]. The piezo-phototronic effect can also be applied to conventional logic devices. Wang et al. designed strain-gated transistors and light-strain-gated transistors to process mechanical and optical stimuli on the devices into electronic controlling signals by tuning the Au/ZnO NWs Schottky barrier. Additionally, inverter, NAND, AND, NOR, OR, and XOR gates with good rectifying behaviors have been fabricated [250]. Moreover, in the field of photocatalysis, a plasmonic bimetallic ZnO nanorod array (Au/ZnO/Pt) can be used for improving catalysis due to the piezo-phototronic effect [251]. It also improves the collection of mechanical energy. ZnO nanorod arrays with an enhanced piezo-phototronic effect can also be used for harvesting low mechanical energy [252]. These applications all show the huge potential of the piezo-phototronic effect.

## 5. Summary and Perspective

This review summarizes the research progress on the modulating role of piezotronic and piezo-phototronic effects on ZnO NW-based semiconductor devices with different structures. The main content is divided into three aspects: the first part introduces the growth method of ZnO NWs, including n-ZnO NWs and p-ZnO NWs, as well as NW arrays with vertical and horizontal structures. The second part introduces the theoretical basis of the piezotronic effect and its applications in the fields of sensors, biochemistry, energy harvesting, and logic operations based on piezoelectric gated transistors. The third part presents the theoretical basis of piezo-phototronics and its modulation effects on photodetectors, LEDs, and solar cells. Additionally, the specific modulation mechanism was discussed in detail by analyzing the energy band diagram. These modulation effects are possible because materials such as a ZnO wurtzite structure have both semiconductor properties and piezoelectric properties, which can not only absorb UV light but also use the piezoelectric potential generated by strain to modulate photogenerated carriers. This will modulate and control the performance of the device. However, although great progress has been made in using ZnO as a piezoelectric semiconductor to study the modulation of the piezotronic and piezo-phototronic effects, there are still many challenges to be overcome, such as those pertaining to stability, selectivity, speed, and versatility. Therefore, based on the current research progress, the study of new piezoelectric semiconductor materials and device structures has great potential for developing piezotronics and piezo-phototronics in energy harvesting and utilization applications. 

The first of these applications is material research. In addition to ZnO, especially ZnO NWs, other wurtzite semiconductors such as GaN, CdS, and InN and other novel materials that have piezoelectric and semiconductor properties simultaneously should be explored as much as possible. The research and utilization of the piezoelectric properties of these materials and the coupling characteristics of the photoexcitation process can also form a new field of study. It is crucial to improve the performance of devices by studying new material-preparation methods to reduce the lattice damage and defect states of materials to improve crystal quality and carrier mobility. With the deepening of piezoelectric semiconductor material research, it has been found that 2D piezoelectric materials are stronger than 1D piezoelectric materials in many characteristics, and they are also more sensitive to the environment. Therefore, there is still much work to be completed to transfer the research direction from 1D to 2D or to combine the advantages of 1D and 2D materials to prepare high-performance optoelectronic devices. Noticeably, 2D ZnO nanosheets have been explored in recent years as promising candidates in piezotronics and piezo-phototronics. In addition, quantum mechanical effects must not be ignored when studying piezoelectric optoelectronics at the nanoscale, and the combination of quantum physics and piezoelectricity is also an important part of future research.

Second, the piezotronic effect uses the piezoelectric potential as a gate signal to regulate the transport process of carriers in the junction region to study and apply novel electronic devices. The piezo-phototronic effect uses the piezoelectric potential to control the process of carrier generation, separation, transmission, and recombination and then modulate the performances of optoelectronic application devices, such as photodetectors, solar cells, and LEDs. The regulation mechanism is mainly the adjustment of the piezoelectric charge introduced by mechanical strain on the energy band structure. Positive piezo-charges bend the energy level downward, and negative piezo-charges bend the energy level upward. Therefore, by analyzing the interface structure and corresponding energy band diagram formed between different materials, efficient utilization of a suitable device structure to introduce piezo-charges to adjust the interface energy band structure, reduce the electron barrier or hole barrier, and improve the transmission efficiency of photogenerated electron–hole pairs is very important to enhance the performance of optoelectronic devices. Despite the traditional rigid Si substrates compatible with common CMOS processes, optoelectronic device structures based on flexible substrates should also be valued. Simultaneous application of pressure and tension on a flexible substrate introduces piezo-charges of opposite polarity, which enables diametrically opposite modulations. At the same time, the conversion of mechanical signals to electrical signals is the core of self-driven electronic devices, so flexible optoelectronic devices are an important research direction in the fields of sensors and human–computer interaction in the future. However, there is a problem. Flexible electronic devices are prone to mechanical fatigue when repeated strain is applied, and irreversible damage will be caused if the strain exceeds the load. Therefore, there is also much research work to be completed to address the stability issue.

Third, the piezo-phototronic effect uses the piezoelectric potential as a gate to change the performance of optoelectronic devices. The flow of photogenerated carriers can be indirectly controlled by mechanical signals. In other words, a mechanical signal that can deform the piezoelectric semiconductor material can be used as a gate. Therefore, wind, water, and human motion can all be used as gate to regulate optoelectronic devices’ performance by adjusting and adopting proper device structures and material properties. This helps to fully use clean energy, which should be a critical research direction in energy harvesting. For example, we can infer the strength of the mechanical effect of seawater on the device through the brightness of the light emitted by the optoelectronic device and then provide a reference for ocean engineering research. In addition, the most important factor is improving energy-conversion efficiency. There are many interferences in the application, so improving the device’s stability and anti-interference properties is important.

Last, the piezo-phototronic effect can also be coupled with other effects to further enhance performance. For example, the coupling of the piezo-phototronic effect and the pyroelectric effect, ferroelectric effect, flexoelectric effect, and electromagnetic effect should all receive attention. By rationally adjusting the device structure and material parameters, the coupling of different modulation effects significantly improves the device’s performance. There are still many modulation effects that we have discovered but have not been cross-studied or undiscovered and are still waiting for us to explore and investigate. These research works have great significance in the scientific research and practical applications of piezotronics and piezo-phototronics.

## Data Availability

No new data were created or analyzed in this study. Data sharing is not applicable to this article.
